# Cardiovascular diseases in mega-countries: the challenges of the nutrition, physical activity and epidemiologic transitions, and the double burden of disease

**DOI:** 10.1097/MOL.0000000000000320

**Published:** 2016-06-14

**Authors:** Simon Barquera, Andrea Pedroza-Tobias, Catalina Medina

**Affiliations:** National Institute of Public Health, Cuernavaca, Morelos, Mexico

**Keywords:** dyslipidemias, mega-countries, nutrition transition, obesity, physical activity

## Abstract

**Purpose of review:**

There are today 11 mega-countries with more than 100 million inhabitants. Together these countries represent more than 60% of the world's population. All are facing noncommunicable chronic disease (NCD) epidemic where high cholesterol, obesity, diabetes, and cardiovascular diseases are becoming the main public health concerns. Most of these countries are facing the double burden of malnutrition where undernutrition and obesity coexist, increasing the complexity for policy design and implementation. The purpose of this study is to describe diverse sociodemographic characteristics of these countries and the challenges for prevention and control in the context of the nutrition transition.

**Recent findings:**

Mega-countries are mostly low or middle-income and are facing important epidemiologic, nutrition, and physical activity transitions because of changes in food systems and unhealthy lifestyles. NCDs are responsible of two-thirds of the 57 million global deaths annually. Approximately, 80% of these are in low and middle-income countries. Only developed countries have been able to reduce mortality rates attributable to recognized risk factors for NCDs, in particular high cholesterol and blood pressure.

**Summary:**

Mega-countries share common characteristics such as complex bureaucracies, internal ethnic, cultural and socioeconomic heterogeneity, and complexities to implement effective health promotion and education policies across population. Priorities for action must be identified and successful lessons and experiences should be carefully analyzed and replicated.

## INTRODUCTION

Mega-countries are defined as countries with a population of at least 100 million inhabitants. Currently, there are 11 nations with this condition: China, India, USA, Indonesia, Brazil, Pakistan, Nigeria, Bangladesh, Russia, Japan, and Mexico (in descending order). In addition, for this study we included three countries with population above 90 million inhabitants that might become eventually mega-countries; Philippines, Ethiopia, and Vietnam [1–3]. This group of highly populated countries is experiencing different stages of the epidemiologic, physical activity, and nutrition transitions and has heterogeneous sociodemographic, economic, and cultural conditions [4^▪▪^]. However, they have also some common characteristics that make this classification useful for analysis from the health policy perspective to identify opportunities for prevention and control of noncommunicable chronic disease (NCDs). Among these, some of the most obvious are: large populations in which even small increases in the prevalence of diseases, have a major impact on thousands of inhabitants, the health system and the national economy, complex bureaucracies which make difficult the coordination of multisectorial policy efforts, big markets, making them a priority target of multinational companies selling tobacco, alcohol, sugar-sweetened beverages (SSB), ultra-processed food, motorized vehicles, TVs, and other products associated with unhealthy lifestyles, overall poor regulatory actions to reduce the impact of the obesogenic environment in health, strong marketing and lobbying of junk food and SSB companies to promote their products and protect their business from regulation and taxation, and participation in trade agreements which are usually decided without considering health implications [2,3,5–10].

During the last 3 decades, a number of factors such as economic growth, urbanization, trade agreements, and improvements in technology have resulted in an epidemiologic physical activity and nutrition transition, which is occurring at a different pace in mega-countries [11–14,15^▪▪^,^16^,^17^]. On the one hand, there are countries at a stage of transition in which a small percentage of urbanization is observed and where undernutrition, high infant mortality rates, low life expectancy, and infections are still major public health problems. However, because of the size of their population, the relatively small but increasing prevalence of NCDs, including cardiovascular diseases (CVDs) and diabetes, are a major health and economic burden, countries in which obesity and NCDs are the main health problem, and countries facing an important double burden of both undernutrition and obesity and NCDs. Although some middle-income countries are experiencing a transition with important progress reducing the undernutrition prevalence, in the mega-country group there are more countries who will transition first to higher prevalences of obesity before controlling undernutrition [18–21]. Recently, low and middle-income countries have experienced an important shift reaching obesity prevalences similar to those of developed countries [22–24,25^▪^] and while the increase in obesity has slowed down in the latter, there are no success stories on efforts to reduce the prevalence of this disease until now [26^▪▪^]. Developed countries have been able to decrease mortality attributable to some risk factors such as high cholesterol, glucose, and blood pressure (BP), but low and middle-income countries are showing alarming increases [25^▪^,^27^^▪▪^,^28^]. A number of factors have contributed to the success in more developed countries, including the combined effect of decreased exposure to tobacco and alcohol consumption, improvements in diet (such as reductions in sodium, fat, and sugar intake), effective investments in preventive programs, physical activity promotion, increased availability of clean potable water, better detection of high-risk populations, improved treatment of CVD and risk factors, and diverse technological developments such as statins, antihypertensive and other drugs, and cardiovascular surgery procedures [14,29,30^▪▪^,^31^,32^▪^,^33^,^34^,35^▪^,^36^–^38^]. However, these improvements have only been able to reduce cardiovascular mortality in Central and Western Europe; during the last decades, an absolute increase has been observed in the global rates of cardiovascular mortality [27^▪▪^].

The purpose of this study is to describe some socioeconomic and demographic characteristics of this heterogeneous group of countries, the current status of the epidemiologic, physical activity, and nutrition transition, the double burden of disease and the challenges to control the rising NCD epidemic. 

**Box 1:**
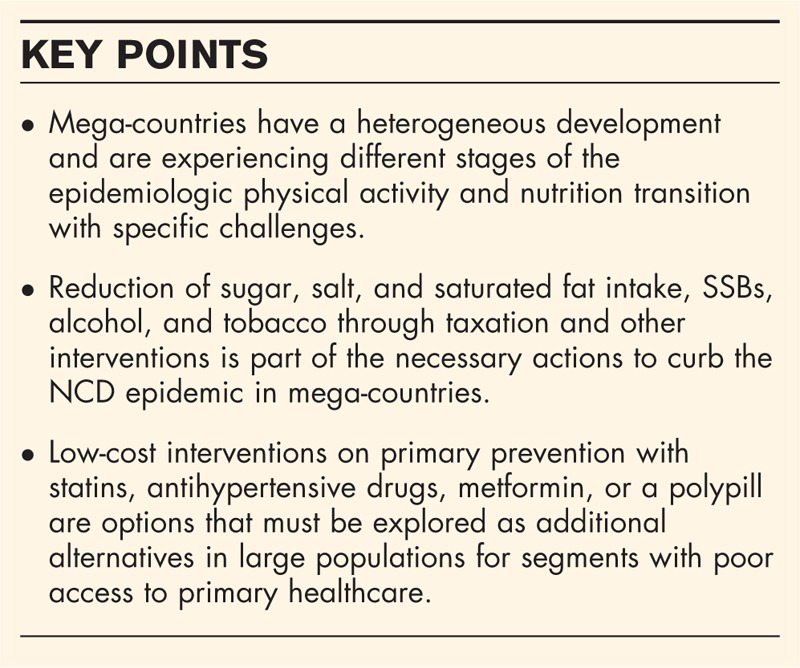
no caption available

## METHODS

For the current analysis, we considered the 11 mega-countries (countries with a population of at least 100 million inhabitants), which are in descending order: China, India, USA, Indonesia, Brazil, Pakistan, Nigeria, Bangladesh, Russia, Japan, and Mexico. In addition, we included three countries with population above 90 million inhabitants that might eventually become mega-countries: Philippines, Ethiopia, and Vietnam [39].

### Socioeconomic and demographic information

We obtained from the World Bank data site, the most recent international socioeconomic indicators available to characterize this group of countries [1]. We included gross domestic product (GDP) ‘per capita’ (2014), Gini index [a proxy measure of distribution of income within a country where 0 represents perfect equality whereas 100 implies perfect inequality (2010), except Brazil, India, and Philippines (2009) and Japan (2008)], annual population growth percentage (2014), life expectancy at birth (2013), urban population percentage (2014), physicians per 1000 inhabitants (2010), and percentage of literacy rate among population older than 15 years of age (2007–2011). We included some indicators of health and education infrastructure; health expenditure expressed as percentage the GDP (2014), health expenditure per capita (2014) and pupil/teacher ratio (2012–2013) except for Nigeria (2010) and Bangladesh (2011).

### Human development index

The index was developed by the United Nations Development Programme more than 25 years ago. It is considered a better measure of development than monetary measures such as the GDP per capita [40,41]. It evaluates three dimensions: health and longevity through life expectancy; access to education through average school years, and standard of living through per capita income (Gross National Income per capita). The most recent human development index (HDI) information was used to classify the mega-countries in four levels of development according to United Nations Development Programme: very high (scores ≥0.8); high (scores ≥0.70 and <0.8); medium (scores ≥0.55 and <0.7); and low (scores <0.55) [42,43]. We later consolidated them in two groups: low and middle-HDI, and high and very high-HDI.

### Prevalence of cardiovascular disease risk factors

Standardized prevalences of overweight and obesity (BMI ≥ 25 kg/m^2^) in adults and stunting (< −2 SD of height for age), a chronic undernutrition indicator in children under 5 years of age, were obtained from the WHO Global Health Observatory. In addition, prevalences of high cholesterol (≥5.0 mmol/l; 2008), high BP (SBP ≥ 140 and/or DBP ≥ 90 mmHg; 2014), and fasting blood glucose (≥7.0 mmol/l, 2014), alcohol (2010) and tobacco (2014) consumption (stratified by sex), and physical inactivity (2010) were obtained from the same source [44]. We used the estimated age-standardized sodium consumption (g per day) (2010) [45] and the estimated SSB consumption (servings/day; stratified by sex) in adults 30–40 years [46^▪▪^].

### Noncommunicable chronic diseases mortality and policy efforts

We used Global Burden of Disease data on percentage of age-adjusted mortality per 100 000 inhabitants during 2013, and the annual percentage change of age-standardized mortality from 1990 to 2013 to evaluate its association to country HDI. Finally, we described policy efforts to curve the NCD epidemic among mega-countries using available systematic reviews from the World Health Organization Global Health Observatory [44], the Global Observatory for Physical Activity [47] and the World Cancer Research Fund Nourishing framework site [48,49], in addition to a number of global and local country reports [50,51^▪^,^52^–^60^].

## RESULTS

Table 1 summarizes the basic economic and sociodemographic information of 14 countries included in this analysis (the 11 mega-countries plus three countries with population above 90 million inhabitants). All analyzed countries belong to three continents, Asia (9), America (3) and Africa (2). There is a very wide range of gross domestic product *per capita* (GDPc) from $46 405.2 in the USA to $315.8 in Ethiopia (2014). Similarly, the range of HDI goes from the highest in the USA (0.91) to the lowest in Ethiopia (0.44). Using the United Nations Development Programme HDI categories there were three countries with low HDI (Pakistan, Nigeria and Ethiopia), five countries with medium HDI (Indonesia, Philippines, Vietnam, India, and Bangladesh), four countries with high HDI (Russia, Brazil, Mexico, and China) and two with very high HDI (USA and Japan). Once the HDI is adjusted for inequality, all country scores are lower, being Brazil, Nigeria, India, Mexico, and Bangladesh the ones in which the HDI reduction is higher. The Gini index, a proxy of income distribution, shows in ascending order that Brazil, Mexico, Philippines, Vietnam, and Nigeria are the countries with the highest income inequality. The percentage annual population growth was negative or less than one digit in five countries: Japan, Russia, China, USA, and Brazil whereas three countries (Nigeria, Ethiopia, and Pakistan), had an annual population growth of more than 2%. The highest life expectancy at birth was observed in Japan (83.3 years), followed by USA (78.8 years), and Mexico (76.5 years), the lowest one was observed in Nigeria (52.4 years), followed by Ethiopia (63.4 years) and Pakistan (66.0 years). Among these countries there are three with more than 80% urban population (Japan, USA, and Brazil), whereas there are five in which urban population is less than 40% (Ethiopia, India, Vietnam, Bangladesh, and Pakistan). USA, Japan and Brazil had the highest health expenditure; USA, Indonesia, and Japan had the lowest pupil/teacher ratio.

**FIGURE 1:**
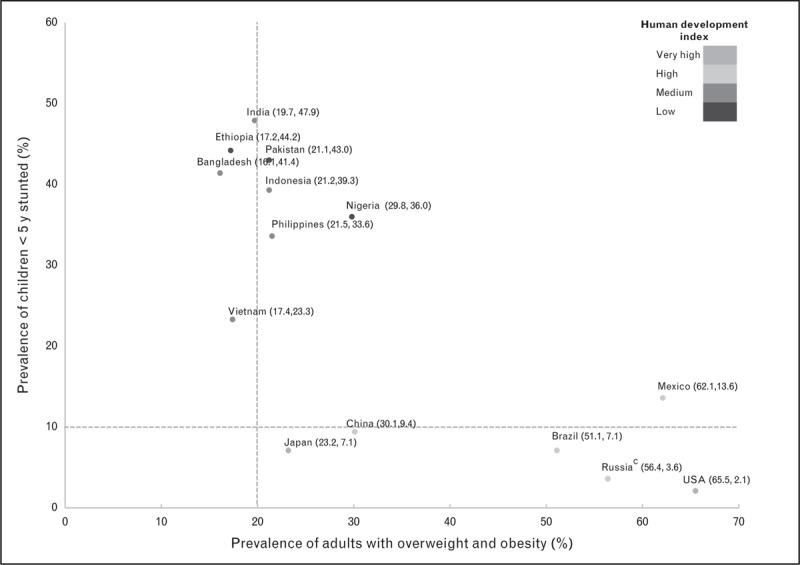
Double burden of malnutrition in mega-countries: coexistence of stunting^a^ and overweight/obesity^b^. ^a^Stunting prevalence: height-for-age z scores less than 2 SD, obtained by the Global Health Observatory of the WHO, 2010-2011, except for India (2005–2006) and Brazil (2006–2007). ^b^Overweight and Obesity prevalence: BMI at least 25 hg/m^2^, obtained by the Global Health Observatory of the WHO, 2010. ^c^Russia prevalence of stunting is not national representative of the under 5-year population.

To explore the level of coexistence of less than 5-year children stunting and adult overweight and obesity by country, a proxy of a phenomenon known as the double burden of malnutrition, a plot was constructed with the intersection between the national prevalence of both conditions (Fig. 1). All low/middle-HDI countries had stunting prevalences more than 20%; within this group India, Ethiopia, Pakistan, Bangladesh, and Indonesia had prevalences more than 40%. Only four mega-countries have stunting prevalences less than 10%: USA, Brazil, China, and Japan and four mega-countries have overweight/obesity prevalence under 20%: Ethiopia, Bangladesh, Vietnam, and India. Among the countries with the highest prevalence of overweight/obesity, Mexico has the highest prevalence of stunting. Similarly, among the countries with the highest prevalence of stunting, Nigeria has the highest prevalence of overweight/obesity being these countries the ones with higher double burden of malnutrition. China and Japan had the lowest combined prevalences of stunting and overweight.

**FIGURE 2:**
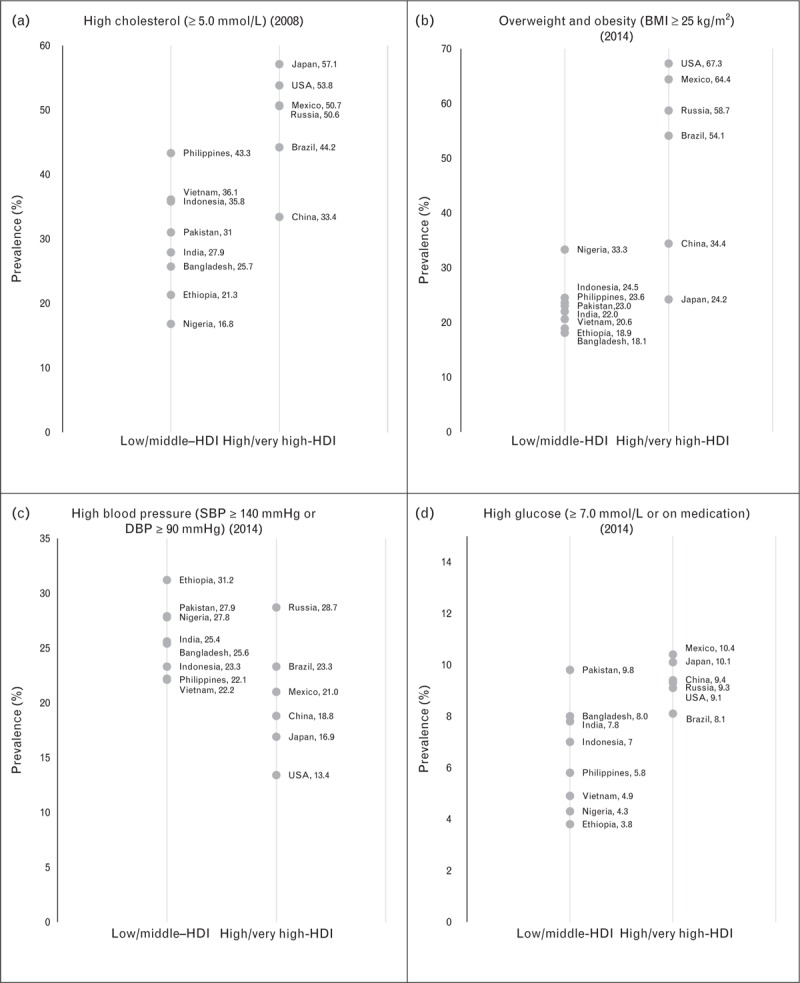
Prevalence of cardiometabolic risk factors in mega countries by HDI category. HDI, human development index. Data obtained by the Global Health Observatory of the WHO.

Figure 2 groups the prevalences of high total cholesterol, overweight and obesity, high BP, and elevated fasting blood glucose by country divided in two groups by HDI classification (low/middle and high/very high HDI). In Japan, USA, Mexico, and Russia more than 50% of the population had high cholesterol levels, whereas in India, Bangladesh, Ethiopia, and Nigeria the prevalences were less than 30%. Overall, the prevalence of cholesterol and overweight/obesity is higher in countries in the high/very high HDI group. The three countries from the American continent (USA, Mexico, and Brazil), together with Russia are the ones with the highest prevalence of overweight and obesity with prevalences more than 50%, whereas the rest of the countries were less than 35%. On the other hand, the prevalences of high BP were higher in the low and middle-HDI group of countries whereas three countries had prevalences less than 20%; USA, Japan, and China. Finally, most of the countries had a prevalence of high blood glucose between 7 and 10%; Mexico and Japan were more than 10% whereas Vietnam, Nigeria, and Ethiopia had prevalences less than 5%.

When comparing risk factors for NCDs among mega-countries, the ones with the highest alcohol consumption were Russia, Japan, and USA with prevalences more than 15%. Five out of six countries in the high/very high-HDI group had prevalences more than 10%, only China had prevalence less than 10%. In contrast, with the exception of Nigeria (7%), all other low/middle-HDI mega-countries had prevalences less than 3%. Japan and USA had a physical inactivity prevalence above 30% whereas in India and Russia it was less than 15%. Tobacco is consumed more by men than by women. The highest tobacco consumption prevalence in men was observed in Indonesia (76.2%) and Russia (59.0%), whereas in women it was observed in Russia (22.8%), USA (15.0%), and Brazil (11.3%). Sodium intake appears to be higher in Asian mega-countries such as Japan, China, Vietnam, Philippines, and Russia. SSB servings per day were higher in Mexico followed by the USA in men and women, and were more consumed among high/very high-HDI countries (Table 2).

**FIGURE 3:**
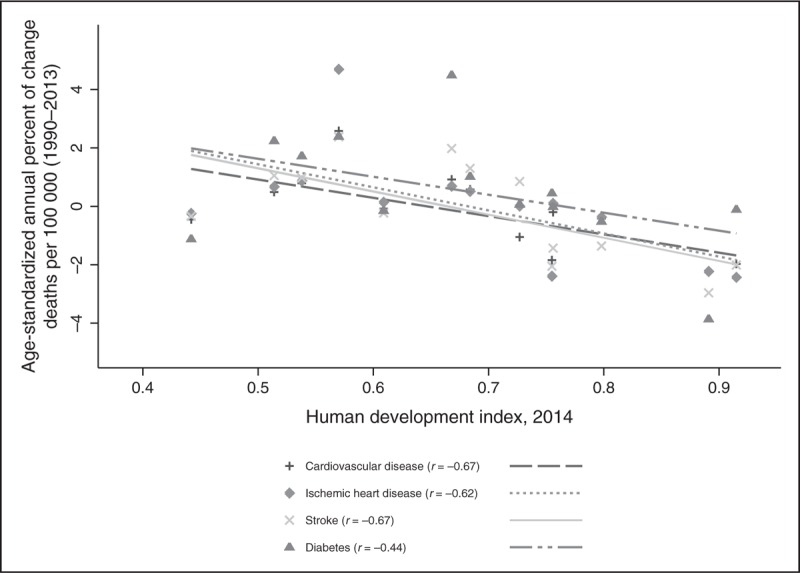
Annual percentage of change of cardiovascular disease, ischemic heart disease, ischemic stroke and diabetes mortality per 100 000 inhabitants (1990–2013) by human development index (2014). Data obtained by Institute for Health Metrics and Evaluation. GBD Compare. Seattle, WA: IHME, University of Washington, 2016. *r*, correlation coefficient.

In Fig. 3, we present the annual percentage change of CVD, ischemic heart disease, ischemic stroke, and diabetes mellitus deaths 1990–2013. The four mortality causes show significant negative correlations to the HDI by megacountry.

## DISCUSSION

Our analysis describes characteristics of 14 mega-countries in which 64.8% of the global cardiovascular mortality occurs. This represents more than 11.2 million deaths per year. These countries have a heterogeneous development and are experiencing different stages of the epidemiologic, physical activity, and nutrition transition. Most of them are middle-income and have a low and middle-HDI. In these countries, the transition to an epidemiologic panorama dominated by NCDs is taking place, whereas high rates of undernutrition are still occurring. This double burden should be addressed with well coordinated intersectorial policies that allow access to traditional diets of better quality without shifting to diets rich in caloric-beverages, ultra-processed foods and high in sugar, fat, and salt [61–63]. Special attention to the promotion of breast-feeding and healthy weaning practices must be in place not only to protect from undernutrition but also from obesity later in life [64]. Some lessons from past experiences in developed countries can be helpful, in particular those of USA and Japan that share the complexities of large populations [14,65]. The recent emphasis on active living and healthier diets using mostly basic foods, avoiding junk food, fast food, and SSBs observed in these countries can be seen as an important step toward healthier lives and could be imported or reinforced in other mega-countries in which the nutrition transition to unhealthy diets is not yet generalized [66,67]. Also, early detection, adequate treatment and other interventions to control risk factors such as high BP, cholesterol, and sugar must be analyzed, implemented with adequate monitoring and evaluation systems. Primary prevention using statins, antihypertensive drugs, or a polypill have showed promising results, and investments on exploring this option should be a priority in countries in which NCDs are increasing and large segments of the population have poor or no access to primary healthcare services [68^▪^,69^▪^,^70^,^71^^▪▪^,^72^–^74^]. Most mega-countries have developed efforts to reduce consumption of tobacco, alcohol, and salt, and to promote appropriate breast-feeding practices and physical activity (Table 3) [13,14,15^▪▪^,^16^,^47^–^49^,51^▪^,^52^–^55^,^57^–^60^,^75^–^77^]. Among the most effective, taxes to tobacco, alcohol, and SSBs have demonstrated to reduce consumption of these unhealthy products and to be a powerful public health tool to complement other strategies to reduce NCDs [4^▪▪^,51^▪^,^52^–^54^,^78^,^79^^▪▪^,^80^–^90^]. Important evidence has been disseminated in the last years on the benefits of salt reduction to reduce CVDs [75,91–95]. In addition, regulation to decrease the use of other unhealthy ingredients such as sugar and saturated/trans fats, to improve food labels to help consumers do healthy choices, to control marketing to children, and to promote physical activity has been recognized as part of the necessary interventions to maximize the opportunity of people adopting healthy lifestyles [10,96–100,101^▪^,^102^,^103^,^104^^▪▪^,^105^–^119^].

These options are particularly important in middle-income countries, where the burden of NCDs is very high and there is an urgent need to identify complementary policies to achieve positive results whereas education, dissemination, promotion and other healthy behavior-oriented efforts, and longer term policies start to generate positive results too (Table 3 summarizes some of the main policy efforts to curve the NCDs epidemic in mega-countries).

Overall, it is possible to organize the mega-countries in three groups by transition stage. These stages are much correlated to diverse measurements of development: *Group 1.* The low/middle-HDI group has low GDPc, is mostly rural, and has a high prevalence of chronic undernutrition and very low prevalences of overweight and obesity. *Group 2*. The high HDI group is composed by four middle-income emerging economies. These countries have an epidemiologic panorama dominated by NCDs and have experienced rapid transitions in the last 3 decades in which most of them went from closed to open economies with international free trade agreements, rapid urbanization, and dramatic changes in lifestyles and food systems. Finally, *Group 3* is composed by the two developed mega-countries with very high-HDI, which are still facing important NCDs but show important progress in a number of indicators. These countries have more than four times the GDPc of any country in *Group 2,* the highest health expenditure per capita, are almost completely urban with very low rates of undernutrition. However, these nations are also in an important need of alternative models to accelerate the prevention and control of NCDs.

Although low/middle-HDI countries in general will have a transition similar to the one experienced by high/very high countries, some characteristics might differ. For example, a number of countries such as Bangladesh, Ethiopia, Nigeria, Pakistan, and Indonesia have a predominant Islam religion where the practice of alcohol consumption is forbidden and therefore cirrhosis because of alcohol use is very low. The poor regulation in low and middle-HDI countries has allowed an important rise in tobacco consumption that has migrated to these nations when more developed ones installed strong regulations on marketing, labeling as well as tobacco taxes. Special attention must be placed in the generalized tobacco consumption in Indonesia where in addition 90% of active smokers report so in the house exposing other family members to secondhand smoke. International experiences could allow this country to overcome this unhealthy practice. All mega-countries report tobacco taxation, however, the total amount of tax is remarkably low in Ethiopia and Nigeria (<21%). Seven mega-countries have a total tax more than 60%; Bangladesh, Pakistan, India, and Philippines in the low/middle HDI group and Brazil, Mexico, and Japan in the high/very high HDI group. In addition, taxes are common in alcoholic beverages. Other policies and regulations such as legal minimum age for tobacco and alcohol, or designated areas for consumption have been implemented. Similarly a number of mega-countries are considering soda taxes as a potential option to decrease consumption. Mexico has adopted a 10% excise tax and a similar measure has been approved in some cities of USA [54,79^▪▪^].

Although most low/middle-income mega-countries have a relatively low prevalence of overweight and obesity, the WHO recommends the use of lower BMI cut points for Asian population as there is evidence of higher prevalences of diabetes and CVD at lower BMI levels [120]. Japan, with low obesity levels, has a high prevalence of hypercholesterolemia. It has increased with the epidemiologic/nutrition transition since the 1960s and currently the highest among the mega-countries. Similarly, the prevalence of high blood glucose in this country is the second highest. This reflects the need to intensify actions to prevent and control emerging risk factors while maintaining successful efforts to decrease others such as high BP [121].

**FIGURE 4:**
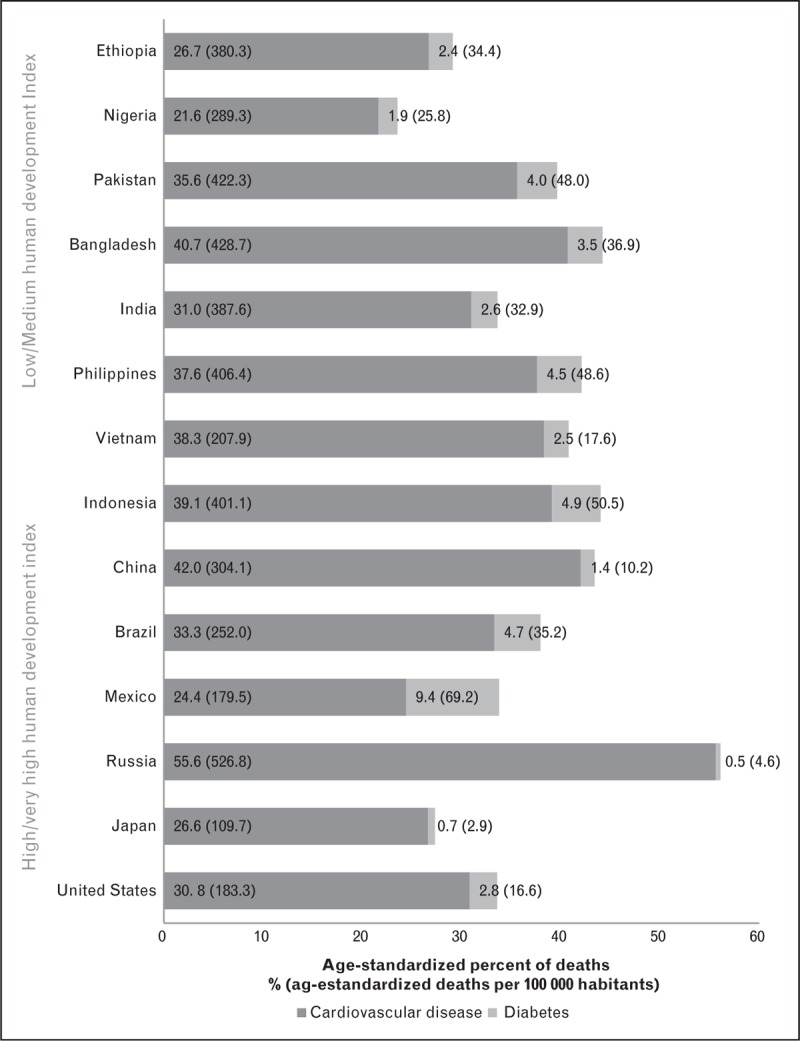
Percentage of deaths attributable to cardiovascular disease and diabetes in mega-countries (2013). Data obtained by Institute for Health Metrics and Evaluation (IHME). GBD Compare. Seattle, WA: IHME, University of Washington, 2016.

The highest prevalence of overweight and obesity in the world is located in the American continent [26^▪▪^]. The two most obese mega-countries are also the ones with the highest SSBs number of servings per day [46^▪▪^]. SSBs are not only associated with obesity but also with diabetes, dyslipidemias, metabolic syndrome, CVDs, and dental health among other health problems [29,122–128]. Russia and Mexico are the mega-countries with the largest percentage of mortality because of CVD and diabetes, respectively. Bangladesh has the highest CVD and diabetes-aggregated mortality among the low/middle-HDI group (Fig. 4).

Finally, it is important in large economies to consider the implications of trade and other macro policies on individual behavior. Shifts in the food system, including prices, distribution, accessibility and quality occur, as consequences of macro policies such as trade, subsidies/incentives and agricultural policies. This in turn, tends to have effects on population health that must be considered by policy makers as one of the components of the decision analysis [7,8,129,130].

## CONCLUSION

The annual percentage change (1990–2013) of CVD, ischemic heart disease, ischemic stroke, and diabetes mortality shows a significant negative trend by HDI of the mega-countries, suggesting that lower HDI mega-countries are shifting to higher mortality by these causes whereas higher HDI are stabilizing. The characteristics of the transition has been described and analyzed and the available information must allow the health and policy-planning sector to identify priorities to diminish the burden of disease. Table 4 summarizes characteristics, expected epidemiological panorama and priorities for low/middle, high/very high-HDI mega-countries.

## Acknowledgements

None.

### Financial support and sponsorship

The work was carried out with support from the International Development Research Centre (INFORMAS project no. 107–731-001). Additional funding was provided by NIH-Fogarty RO3 project no. TW009061 and an unrestricted research grant from Sanofi-Aventis to the National Institute of Public Health.

### Conflicts of interest

There are no conflicts of interest.

## REFERENCES AND RECOMMENDED READING

Papers of particular interest, published within the annual period of review, have been highlighted as:

▪ of special interest
▪▪ of outstanding interest

## Figures and Tables

**Table 1:** Socioeconomic characteristics of mega-countries

	Low middle HDI								High very high HDI					
Indicator	Ethiopia	Nigeria	Pakistan	Bangladesh	India	Philippines	Vietnam	Indonesia	China	Brazil	Mexico	Russia	Japan	USA
Human Development Index, 2014	0.44	0.51	0.54	0.57	0.61	0.67	0.67	0.68	0.73	0.76	0.76	0.80	0.89	0.91
Category Human Development Index	Low	Low	Low	Medium	Medium	Medium	Medium	Medium	High	High	High	High	Very high	Very High
Population (million), 2014	96.9	177.5	185.0	159.1	1,295.3	99.1	90.9	254.5	1,364.3	206.1	125.4	143.8	127.1	318.9
GDPc (2014) (constant USD 2005)	315.8	1,098.0	813.7	747.4	1,233.9	1,662.1	1,077.9	1,853.8	3,862.9	5,880.6	8,521.9	6,843.9	37,595.2	46,405.2
Gini Index (2010)^a^	33.2	43.0	29.6	32.0	35.6	42.9	42.7	33.9	42.1	53.9	48.1	40.9	32.1	40.5
Inequality-adjusted Human Development Index, 2014	0.31	0.32	0.38	0.40	0.43	0.55	0.54	0.56	–	0.56	0.59	0.71	0.78	0.76
Annual population growth (%), 2014	2.51	2.66	2.10	1.21	1.23	1.59	1.07	1.26	0.51	0.89	1.32	0.22	−0.16	0.74
Life expectancy at birth 2013 (years)	63.4	52.4	66.0	71.3	67.7	68.1	75.8	68.7	75.4	74.1	76.5	71.1	83.3	78.8
Urban population (%), 2014	19.0	46.9	38.3	33.5	32.4	44.5	33.0	53.0	54.4	85.4	79.0	73.9	93.0	81.5
Physician (per 1000 people), 2010	0.02	0.40	0.83	0.30	0.65	–	1.22	0.29	1.80	1.76	1.96	4.31	2.30	2.42
Adult literacy rate population 15+ years, 2007–2011	39.0	51.1	55.4	59.7	69.3	95.4	93.5	92.8	95.1	90.4	93.1	99.7	–	–
Health expenditure per capita (current USD$), 2014	26.6	117.5	36.2	30.8	75.0	135.2	142.4	99.4	419.7	947.4	677.2	892.9	3,703.0	9,402.5
Health expenditure (% GDP), 2014	4.9	3.7	2.6	2.8	4.7	4.7	7.1	2.8	5.5	8.3	6.3	7.1	10.2	17.1
Pupil/teacher ratio (2010-2013)^b^	53.7	37.6 (2010)	42.5	40.2 (2011)	32.3	31.4	18.9	16.1	16.9	21.2	28.0	19.6	16.7	14.5

GDPc, gross domestic product *per capita*. Data obtained by World Bank (2016).

^a^Brazil, India, Nigeria, and Philippines: Gini index 2009; Japan: Gini Index 2008.

^b^Nigeria: pupil/teacher ratio 2010; Bangladesh: pupil/teacher ratio 2010.

**Table 2:** Major risk factors for the development of noncommunicable diseases in mega-countries

	Heavy drinking past 30 days^a^^,^^b^ % (95%CI)	Insufficiently active adults^a^^,^^b^ % (95%CI)	Use of tobacco products in population at least 15 years^a^ % (95%CI)		Estimated sodium intake (g/day)^b^ % (95%CI)	Estimated SSB (servings/day) (30–40 years old) % (95%CI) [46^▪▪^]	
Year	2010	2010	2015		2010 [45]	2010	
Low/middle HDI			Women	Men		Women	Men
Ethiopia	0.6 (0.0–1.2)	18.9 (4.9–50.1)	0.5 (0.2–0.8)	8.9 (6.0–12.1)	2.27 (1.95–2.67)	0.26 (0.14–0.43)	0.28 (0.15–0.47)
Nigeria	7.0 (0.1–8.9)	22.3 (8.5–66.3)	1.1 (0.4–2.0)	17.4 (8.0–28.9)	2.82 (2.51–3.17)	0.37 (0.20–0.61)	0.41 (0.23–0.66)
Pakistan	0.1 (0.0–0.3)	26 (7.2–62)	3.0 (1.8–4.2)	41.9 (29.7–57.3)	3.91 (3.32–4.66)	0.66 (0.35–1.44)	0.73 (0.40–1.20)
Bangladesh	0.0 (0.0–0.2)	26.8 (25.9–27.8)	0.7 (0.4–1.0)	39.8 (30.6–50.1)	3.54 (2.98–4.21)	0.19 (0.10–0.39)	0.21 (0.11–0.37)
India	1.6 (0.7–2.6)	13.4 (12.2–14.8)	1.9 (1.4–2.5)	20.4 (14.5–27.3)	3.72 (3.63–3.82)	0.42 (0.22–0.72)	0.47 (0.26–0.77)
Philippines	1.6 (0.7–2.6)	15.8 (3.6–44.2)	8.5 (6.6–10.8)	43.0 (34.6–53.5)	4.29 (3.65–5.10)	0.56 (0.31–0.94)	0.62 (0.34–1.05)
Vietnam	1.3 (0.4–2.1)	23.9 (16.6–32.9)	1.3 (0.9–1.6)	47.1 (35.7–58.5)	4.59 (3.81–5.46)	0.32 (0.18–0.54)	0.35 (0.20–0.57)
Indonesia	2.4 (1.2–3.6)	23.7 (19–29.1)	3.6 (2.6–4.5)	76.2 (59.5–95.5)	3.36 (3.02–3.76)	0.35 (0.19–0.57)	0.37 (0.21–0.64)
High/very high HDI							
China	7.5 (5.5–9.5)	24.1 (21.7–26.5)	1.8 (1.3–2.2)	47.6 (36.7–58.6)	4.83 (4.62–5.05)	0.07 (0.06–0.08)	0.08 (0.06–0.09)
Brazil	12.2 (9.7–14.6)	27.8 (8–3.9)	11.3 (8.2–14.6)	19.3 (14.6–24.4)	4.11 (4.01–4.22)	0.60 (0.53–0.68)	0.66 (0.58–0.75)
Mexico	10.9 (8.6–13.3)	26 (20.5–32.1)	6.6 (5.2–8.2)	20.8 (16.4–25.3)	2.76 (2.57–2.94)	1.87 (1.36–1.48)	2.03 (1.46–2.72)
Russian Federation	19.3 (16.3–22.3)	9.5 (6.8–12.8)	22.8 (17.6–29.3)	59.0 (46.6–72.5)	4.17 (3.95–4.40)	0.56 (0.32–0.90)	0.62 (0.34–1.01)
Japan	18.4 (15.4–21.3)	33.8 (11.1–71.6)	10.6 (8.0–13.4)	33.7 (25.9–41.6)	4.89 (4.71–5.08)	0.38 (0.32–0.44)	0.45 (0.38–0.51)
USA	16.2 (13.4–19.0)	32.4 (29.8–35)	15.0 (12.1–18.1)	19.5 (15.7–23.6)	3.60 (3.50–3.70)	1.43 (1.25–1.62)	1.65 (1.44–1.88)

^a^WHO Global Health Observatory (2016).

^b^Age-standardized estimates.

**Table 3:** Current policy efforts to curve the non-communicable diseases epidemic among mega-countries

Country	Tobacco^a^	Alcohol^b^	Salt (SI/SR)^c^	SSBs^d^	Breastfeeding^e^	National plan to promote physical activity
Low/middle HDI						
Bangladesh	T: 76%, AVE: 61%, VAT: 15%	LMA: No, VAT: 15%, ET: N/A	Yes/Yes	–	EB: 97.8%, EBF < 6m: 42.9%, BFP: yes	Yes
Ethiopia	T: 18.77%, AVE: 13.9%, VAT: 4.87%	LMA: 18y, VAT: 15%, ET: b50%, w50%, s100%	Yes/–	–	EB: 96%, EBF < 6m: 49%, BFP: yes	N/A
Nigeria	T: 20.63%, AVE: 15.87%, VAT: 4.76%	LMA: 18y, VAT: 5%, ET: b2%, w2%, s2%	Yes/–	–	EB: 97.3%, EBF < 6m: 13.1%, BFP: yes	No
Pakistan	T: 60.70%, SE: 46.17%, VAT: 14.53%	LMA: 21y, VAT: No, ET: N/A	Yes/–	–	EB: 94.3%, EBF < 6m: 37.1%, BFP: –	N/A
India	T: 60.39%, SE: 42.45%, AVE: 1.27%, VAT: 16.67%	LMA: subnational, VAT: No, ET: N/A	Yes/–	Considering tax MUFB	EB: 95.7%, EBF < 6m: 46.4%, BFP: yes	Yes
Indonesia	T: 53.40%, SE: 40.91%, AVE: 4.09%, VAT: 8.40%	LMA: 21y, VAT: 10%, ET: N/A	Yes/Yes	Considering tax	EB: 95.2%, EBF < 6m: 32.4%, BFP: –	Yes
Philippines	T: 74.27%, SE: 63.55% VAT: 10.71%	LMA: 18y, VAT: 12%, ET: N/A	Yes/Yes	Considering tax	EB: 87.7%, EBF < 6m: 34%, BFP: yes	N/A
Vietnam	T: 41.59%, AVE: 32.50% VAT: 9.09%	LMA: 18y, VAT: 10%, ET: b45%, w45%, s45%	Yes/Yes	–	EB: 97.7%, EBF < 6m: 16.9%, BFP: yes	Yes
High/very high HDI						
Brazil	T: 64.94%, SE: 20.87%, AVE: 8.10%, VAT: 25%, OT: 10.97%	LMA: 18y, VAT: N/A, ET: b6%, w6%, s5%	–/Yes	Considering tax MUFB	EB: 96.4%, EBF < 6m: 39.8%, BFP: yes	Yes
China	T: 44.43%, SE: 0.60%, AVE: 29.30%, VAT: 14.53%	LMA: No, VAT: 17%, ET: bN/A, w1%, s2%	Yes/Yes	MUFB	EB: –, EBF < 6m: –, BFP: yes	Yes
Mexico	T: 65.87%, SE: 15.56%, AVE: 36.52%, VAT: 13.79%	LMA: 18y, VAT: 16%, ET: b25%, w25%, s50%	Yes/Yes	Approved tax MUFB	EB: 92.3%, EBF < 6m: 20.3%, BFP: yes	Yes
Russian Federation	T: 47.63%, SE: 23.88%, AVE: 8.50%, VAT: 15.25%	LMA: 18y, VAT: 18%, ET: N/A	Yes/–	–	EB: –, EBF < 6m: –, BFP: yes	No
Japan	T: 64.36%, SE: 56.95%, VAT: 7.41%	LMA: 20y, VAT: 5%, ET: b5%, w5%, s5%	–/Yes	–	EB: –, EBF < 6m: 21% BFP: yes	Yes
United States	T: 42.54%, SE: 37.38% VAT: 5.16%	LMA: 21y, VAT: No, ET: b$3.75, w$0.72%, l$0.2^a^	Yes/Yes	Approved tax in 1 city. Considering tax in other states MUFB	EB: 73.9%, EBF < 6m: 13.6%, BFP: yes	Yes

HDI, human development index; N/A, not available; NCD, noncommunicable diseases.

^a^Tobacco: (taxes on the most sold brand of cigarettes (% of retail price)), (T – total, ADE – Ad valorem excise, SE – Specific excise, VAT – value added tax, OT – Other taxes).

^b^Alcohol (LMA – legal minimum age, ET – excise tax as a percent of the retail price of alcoholic beverages. B – beer, w – wine, s – spirits, l – liquor) ^*^$ per gallon.

^c^Salt (SI – salt iodization, SR – salt reduction).

^d^MUFB – Marketing of unhealthy foods and beverages to children.

^e^Breastfeeding (EB – ever breastfed, EBF < 6m – exclusive < 6 months breastfeeding, BFP – breastfeeding promotion).

**Table 4:** Stages of the nutrition transition and challenges for mega-countries to address the burden of non-communicable chronic diseases^a^

Country group	Characteristics	What could be expected in the next decades?	What priorities must be addressed to diminish the burden of NCDs?
Low-Middle HDI	Mostly rural/labor-intensive work low sedentarism	Increasing urbanization trend. Decrease in physical activity associated with shifts to lower labor-intensive occupations and active transportation.	Raise priority of NCDs in development of national policies
Pattern 3 of the nutrition transition (receding famine) Bangladesh, Ethiopia, Nigeria, Pakistan, India, Indonesia, Philippines, Vietnam	Epidemiologic panorama dominated by under nutrition, but even low prevalence of NCDs represent a significant burden	Decreasing prevalence of stunting. Increasing prevalence of NCDs associated to national growth, trade and shifts in the food systems with low priority and resources from the governments and health sector to respond.	Improve/develop surveillance and monitoring systems for NCDs
	Increasing prevalence trends of NCDs (obesity, high blood cholesterol, blood pressure and blood sugar)	Higher prevalence of CVD at lower BMI levels than in higher HDI megacountries	Implement policies to promote adequate urban planning focused on active communities, active transportation and public transportation
	Elevated population growth	Tobacco consumption becoming major public health problem in particular among males	Disincentives for use of cars. Promotion of physical activity to compensate for increasingly sedentary jobs
	Starchy, high fiber, hydration mostly with water	Increasing consumption of processed foods, sugar-sweetened beverages and fast food	Reinforcement/Implementation of taxes and strict regulation to disincentive tobacco consumption
	Increasing consumption of tobacco	Alcohol consumption not expected to increase in most of these countries due to Muslim religion practices	Promotion and incentives to reinforce/maintain adequate breast-feeding practices
			Policies and incentives to maintain and value traditional foods and diets as the key to adequate nutrition. Guidelines for healthy hydration and disincentives to the development of SSBs industry.
			Introducing/improving nutrition education with emphasis on the unhealthy effects of high added sugar, salt and saturated/trans fats intake
High HDI	Mostly urban/physical activity very low	Increasing urbanization, often without proper urban planning. Decrease in physical activity (due mostly to shifts in labor) and active transportation	Raise priority of NCDs in design of national policies with a multisectoral approach. Develop monitoring systems to evaluate progress in NCDs prevention and control at global, regional and national levels
Pattern 4 of the nutrition transition (rise of noncommunicable diseases) Brazil, China, Mexico, Russia	Epidemiologic panorama dominated by NCDs (obesity, high blood cholesterol, blood pressure and blood sugar)	Decreasing prevalence of stunting. NCDs recognized as the main public health problem, however lack of comprehensive policies to tackle the epidemic	Promote a strong focus on urban planning considering active transportation, walkability and active living. Disincentives such as taxes and regulation for the use of cars and incentives for use of public transportation
	High tobacco, SSBs, fast foods and processed foods consumption. Poor potable water availability	Tobacco consumption remains a major public health problem with increases in consumption by vulnerable groups (young adults, women, low-income, ethnic minorities)	Continuing/improving efforts to reduce tobacco, SSBs and alcohol consumption: taxation, marketing and labeling regulations. Avoid subsidies on unhealthy ingredients/avoid policies that increase availability of sugar, high fructose and other caloric sweeteners, salt and saturated fats
	Poor public transportation, inadequate/insufficient public parks	Increasing consumption of processed foods, sugar-sweetened beverages, fast food	Improve health care response to the rapid rise in NCDs: review and update health care curricula and training, massive education campaigns, early detection, and effective treatment and control
	Lack of adequate regulations to protect children from unhealthy food and beverage marketing	Imported low-cost solutions to control CVD risk factors such as statins, antihypertensive drugs and aspirin will become more available	Develop creative solutions to address de NCDs burden such as: national soda taxation programs, strong healthy lifestyles programs, labeling and marketing regulations, polypill interventions in populations with poor access to health care, etc. Import effective initiatives and technology
	Transnational food companies lobbing against health policy attempts to modify food environment	Trends on implementation of creative national policies to improve food environment (soda tax, ultra processed food marketing and labeling regulation, physical activity promotion)	Develop international trade agreements that include healthy lifestyle considerations
Very High HDI	Mostly urban/physical activity very low however increasing, particularly on higher socioeconomic segments	Urban planning improving, with emphasis on walkability, active living, sustainability and environment.	Strengthen national policies and plans for the prevention and control of NCDs
Pattern 5 of the Nutrition Transition (desired societal/ behavioral change) Japan, USA	Epidemiologic panorama dominated by obesity and NCDs, however, reductions in smoking, SSBs and other risk factors	Stabilization/ saturation equilibrium/ slow reduction of obesity and NCDs	Promote research for the prevention and control of NCDs at global and national levels
	Relatively good public transportation, public parks, and potable water availability. Trends toward the practice of active transportation	Vulnerable groups such as low socio economic sectors, women, adolescents and minorities with higher exposure to unhealthy lifestyles: tobacco consumption, ultra processed foods, SSBs, fast foods, inactive transportation, poor access to incentives for physical activity	Continued improvement of environments to promote healthy lifestyles with emphasis on vulnerable groups (such as low-income population, disadvantaged ethnic groups, women and adolescents), to reduce inequalities in health
	Increasing trend to local, traditional unprocessed foods and healthy hydration	Effective solutions to control some CVD risk factors such as statins, antihypertensive drugs and aspirin widely available at low cost to the vast majority of population	Import effective national policies implemented in High HDI countries such as soda tax, ultra processed front-of-pack labeling, cycling paths, nation wide physical fitness programs, etc.
		Decreasing trend in consumption of processed foods, sugar-sweetened beverages and fast food franchises in particular on higher socioeconomic segments of the population	Control of emerging risk factors must be emphasized (high cholesterol and glucose in Japan; high tobacco consumption and sugar-sweetened beverages consumption in USA).
		Unhealthy products migrating to emerging, less regulated markets in other countries	Priority to low-income population and other disadvantaged minorities and vulnerable groups
			Promote social responsibility and fair trade of companies attempting to sell unhealthy products overseas

CVD, cardiovascular disease; HDI, human development index; NCD, noncommunicable chronic disease; SSB, sugar-sweetened beverage.

^a^Patterns of the nutrition transition according to Popkin (2015).
